# Aggressive Disease Course of Papillary Thyroid Carcinoma with Focal Undifferentiated Component: A Case Report

**DOI:** 10.4274/mirt.38358

**Published:** 2016-09-29

**Authors:** Saima Riaz, Humayun Bashir, Aamna Hassan, Sajid Mushtaq, Arif Jamshed, Ahmad Murtaza

**Affiliations:** 1 Shaukat Khanum Memorial Cancer Hospital and Research Centre, Clinic of Nuclear Medicine, Lahore, Pakistan; 2 Shaukat Khanum Memorial Cancer Hospital and Research Centre, Clinic of Pathology, Lahore, Pakistan; 3 Shaukat Khanum Memorial Cancer Hospital and Research Centre, Clinic of Radiation Oncology, Lahore, Pakistan; 4 Shaukat Khanum Memorial Cancer Hospital and Research Centre, Clinic of Radiology, Lahore, Pakistan

**Keywords:** Papillary thyroid carcinoma, undifferentiated thyroid carcinoma, thyroglobulin, radioiodine therapy, fludeoxyglucose positron emission tomography

## Abstract

We report an aggressive papillary thyroid carcinoma (PTC) with focal undifferentiated component in a 32-year-old female. She had limited disease confined within the thyroid gland at diagnosis. Within 12 months of thyroidectomy and radioiodine ablation, thyroglobulin (Tg) levels were elevated. Second radioiodine ablative dose was given, however, stimulated Tg levels showed an upward trend with negative iodine scan within 12 months. An ^18^F fludeoxyglucose-avid solitary pulmonary nodule that was detected on positron emission tomography/computed tomography scan was resected followed by empiric radioiodine therapy. Within the next 10 months she developed multifocal bone metastases. The multifocal disease was rendered inoperable and treated with external beam radiation. The patient is on follow-up, and the Tg level continues to rise with local disease progression. In a small percentage of patients, PTC behaves as a very aggressive disease despite treatment. Focally undifferentiated thyroid carcinoma is an expression of the extreme end of the spectrum of differentiated thyroid carcinoma.

## Abstract (tr)

Otuz iki yaşında bir kadında saptanan fokal andiferansiye komponentli agresif seyirli papiller tiroid karsinomu sunulmaktadır. Tanı anında tiroid bezi ile sınırlı hastalığı mevcuttu. Tiroidektomi ve radyoiyot ablasyondan 12 ay sonra tiroglobulin (Tg) değerleri yükselmeye başladı. İkinci radyoiyot ablasyon dozu uygulandı, ancak stimule Tg düzeyleri yükselme eğiliminde idi ve 12. ayda iyot sintingrafisi negatif idi. Pozitron emisyon tomografisi/bilgisayarlı tomografi ile [ref:18]18[/ref]F florodeoksiglukoz-avid soliter pulmoner nodül saptandı, rezeke edildi ve ampirik radyoiyot tedavisi uygulandı. Takip eden 10 ay içinde multifokal kemik metastazları saptandı. Multifokal hastalık inoperabl olarak değerlendirildi ve eksternal radyasyon ile tedavi edildi. Hasta halen takip altında olup Tg değeri lokal hastalık progresyonu ile birlikte yükselmeye devam etmektedir. Hastaların küçük bir bölümünde papiller tiroid karsinomu tedaviye rağmen agresif seyirli olabilir. Fokal andiferansiye tiroid karsinomu diferansiye tiroid karsinom spektrumunun en aşırı ucunu temsil etmektedir.

## INTRODUCTION

Thyroid carcinoma has been classified according to the degree of differentiation at two ends of the disease spectrum; the well differentiated and the undifferentiated (anaplastic) carcinomas. There is a definite morphological and behavioral distinction between these two entities. The well-differentiated carcinomas are classified as papillary and follicular carcinomas. At the other end of the spectrum, the undifferentiated group includes anaplastic, insular and other types of carcinomas. Behavior-wise, the papillary and follicular carcinomas are relatively indolent and curable, while the anaplastic variant is highly aggressive (1).

Papillary thyroid carcinoma (PTC) is the most common thyroid malignancy, generally with an indolent clinical course. The overall 5-year relative survival rate has been reported as high as 97.5%, and only a small percentage of papillary carcinomas show aggressive clinical behavior (2).

The aggressive subtypes of PTC include tall cell, columnar cell, diffuse sclerosing variant and hobnail variant. These variants have been associated with higher rates of extra-thyroidal extension, multi-focality, nodal and distant metastasis, recurrence, and resistance to radioactive iodine therapy (3,4). The case we describe here had a discrete undifferentiated focus in the background of classical PTC.

## CASE REPORT

**Figure 1:**
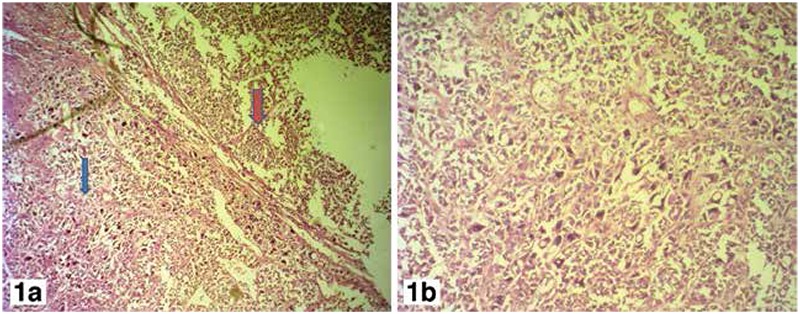
a) The histopathology slide (blue arrow) indicating undifferentiated component in the tumor with large, hyperchromatic and bizarre looking nuclei. The red arrow points towards the conventional papillary carcinoma, b) High power view of the focal undifferentiated component

In September 2010, a 32-year-old female presented for the evaluation of a right thyroid nodule, which has gradually increased in size over the past 5 years. There were no associated compression symptoms. She underwent right lobectomy and isthmectomy on June 3’ 2010. The histopathologic findings were consistent with a neoplastic lesion with vascular invasion. Completion thyroidectomy was performed on June 28’ 2016 and histology showed papillary carcinoma with a focal undifferentiated component (T3 N0 M0) (Figure 1a, b).

In view of the undifferentiated component within a background of PTC, a bone scan and computed tomography (CT) scan (chest, abdomen and pelvis) were acquired for staging. All baseline work-up was negative for metastatic disease. After thyroidectomy she received a radioactive iodine ablative dose of 120 mCi in September 2010. Stimulated thyroglobulin (Tg) levels were 17.4 ng/ml. An iodine avid remnant was detectedin the thyroid bed on post-therapy whole body scan.

**Figure 2:**
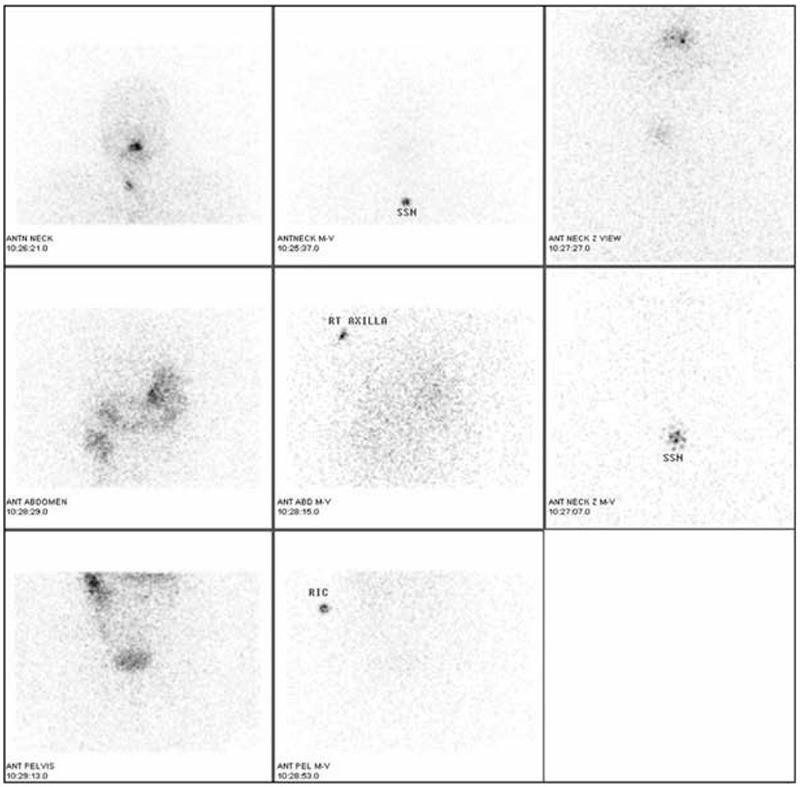
Post-radioiodine therapy whole body spot views showing avid uptake by the residual thyroid tissue. Physiological tracer distribution is detected in the nasopharynx, gut and urinary bladder

At 12 months, stimulated Tg levels were elevated at 109 ng/ml. A diagnostic ^131^I whole body scan showed uptake in the thyroid bed. A second ablative dose of 100 mCi was given in January 2012. Post-therapy scan showed delivery of iodine to the thyroid remnant in the neck (Figure 2).

In February 2013, the patient was clinically symptom free. Stimulated Tg rose to 34 ng/ml. A diagnostic ^131^I scan did not show any abnormal uptake.

**Figure 3:**
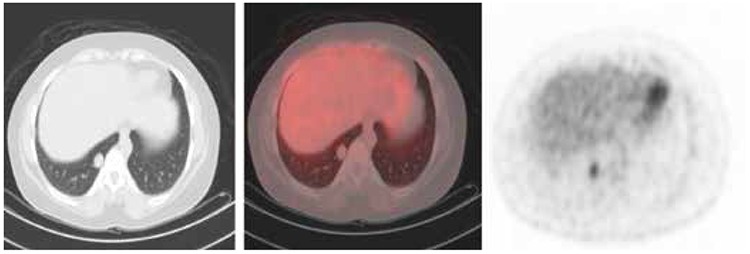
Trans-axial computed tomography, fusion positron emission tomography/computed tomography and positron emission tomography images of the chest showing hypermetabolic, 1.8 cm, round right lower lobe pulmonary nodule with a SUV^max^ of 3.3

To search for occult metastases, a 18F fludeoxyglucose (FDG) positron emission tomography (PET)/CT scan was acquired that revealed a 1.8 cm hypermetabolic, round, right lower lobe pulmonary nodule with a standardized uptake value (SUV_max_) of 3.3 (Figure 3).

In March 2013, the patient underwent a right sided video assisted thoracoscopic surgery and lower lobe nodulectomy. Histopathology was consistent with metastatic papillary carcinoma (2.0 cm), and the resection margin was disease free. An empiric dose ^131^I (150 mCi) was given in June 2013 (stimulated Tg=295 ng/ml).

**Figure 4:**
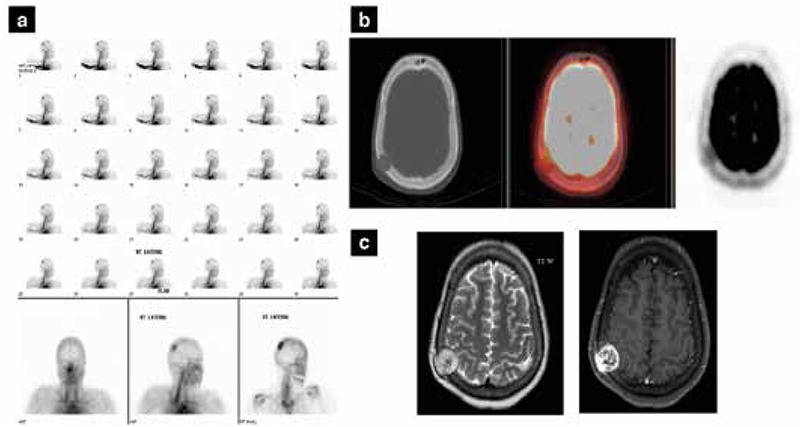
Three phase bone scan of the skull showing increased blood flow, focal hyperemia and osteoblastic activity in the right parietal bone, b) Trans-axial computed tomography, fusion positron emission tomography/computed tomography, and positron emission tomography views of the skull demonstrating a large lytic lesion at the posterior aspect of the right parietal bone, eroding both inner and outer tables with an associated subcutaneous and intracranial (extradural) soft tissue component (SUV_max_ of 4.4). No obvious intra axial lesion is identified, c) Trans-axial magnetic resonance imaging scan of the skull showing a 3 cm right parietal expansile lesion. The mass is T1 hypointense, T2 heterogeneous with avid post-contrast enhancement and associated extra-axial soft tissue involvement on either side of the calvarium

In December 2013, the patient presented with a palpable scalp swelling in the posterior parietal region, that was noticed only 10-12 days ago. On thyroxine, the Tg was 452 ng/ml. A three phase bone scan showed osseous uptake in the right parietal bone (Figure 4a). This lesion was hypermetabolic (SUV_max_ 4.4) on PET/CT scan with erosion of the inner and outer tables of the right parietal bone and an associated subcutaneous and intracranial (extradural) soft tissue component. No obvious intra-axial lesion was identified (Figure 4b).

Magnetic resonance imaging (MRI) scan of the brain confirmed a 3-cm expansile lesion in the right parietal region. The mass was T1 hypointense, T2 heterogeneous with post-contrast enhancement, and with associated extra-axial soft tissue on either side of the calvarium (Figure 4c).

**Figure 5:**
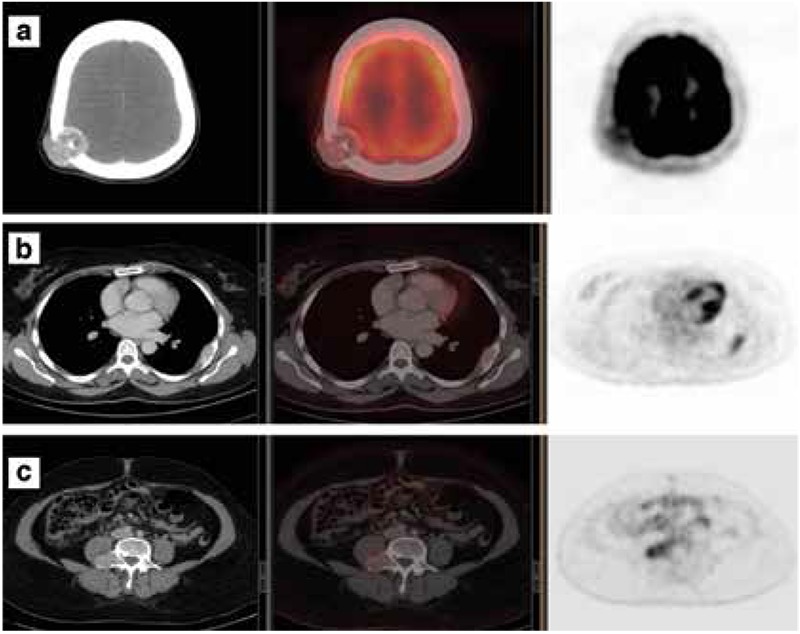
a) Trans-axial computed tomography, fusion positron emission tomography/computed tomography (PET/CT) and PET views of the skull showing metastatic deposit in the right parietal bone postero-laterally with interval progression in size and increase in both intracranial (extra-axial) and subcutaneous soft tissue components, b) Trans-axial computed tomography, fusion PET/CT and PET views of the chest showing a lytic lesion with a large soft tissue component in the 5th left rib postero-laterally which is mildly metabolically active (SUV_max_ of 3.4), c) Trans-axial CT, fusion PET/CT and PET views of the pelvis showing an expansile mass appreciable in the right pedicle and transverse process of the L4 vertebra without intra-spinal extension (SUV_max_ of 4.1)

According to the neurosurgical consultation, surgical removal of the solitary skull metastasis was planned. However, there was a three month delay due to patient logistics, and pre-surgery Tg level rose to 1623 ng/ml. A repeat FDG PET/CT scan revealed progression of the parietal bone metastasis. Additional hypermetabolic, expansile, lytic lesions (with soft tissue component) were identified in the 5th left rib postero-laterally (SUV_max_ of 3.4), and in the right pedicle and transverse process of the L4 vertebra without intra-spinal extension (SUV of 4.1) (Figure 5). In retrospect, these lesions were also faintly visible on the baseline FDG PET/CT scan.

A CT-guided biopsy of the soft tissue component of the most accessible L4 vertebra lesion confirmed metastatic PTC. The patient underwent palliative radiotherapy to the skull, left rib and the lumbar vertebra with 20 Gy/5 fractions. She is on follow-up with complaints of mild pain along the rib and the L4 lesion that require PRN oral analgesics. FDG PET/CT performed 6 months post-XRT showed local progression at the three lesion sites, with no new identifiable lesion.

## DISCUSSION

PTC is the most common thyroid malignancy and represents 75 to 85 percent of all thyroid cancers. PTC is frequently found in women of the 20 to 55-year age group (5).

Papillary carcinoma usually has an indolent course rarely showing aggressive behavior. This small percentage has been referred as “real carcinomas“ of the thyroid, bringing a challenge to management (6).

The undifferentiated anaplastic carcinoma comprises only 1.7% of thyroid cancers. It is an extremely malignant neoplasm. Its incidence typically peaks at the 6-7^th^ decade of life with a median survival of 3 months following diagnosis. Despite the use of multimodality treatment combining surgery, external beam radiation and chemotherapy, long term survival is possible in only selected patients (7,8,9).

Undifferentiated (anaplastic) cancers commonly metastasize to the regional lymph nodes and lungs. Because of its aggressive behavior, all anaplastic thyroid tumors are classified as stage IV regardless of tumor size, location or metastasis (10).

Differentiated and undifferentiated variants may arise simultaneously in neoplastic lesions. Perri et al. (11) and Foote et al. (12) showed that in certain cases a portion of an otherwise well-differentiated carcinoma may containun differentiated (anaplastic) carcinoma. This has been observed in middle-aged or elderly patients, with grave prognosis. A small focus measuring only a few millimeters in diameter may have little effect on the long-term survival. However, in some patients, this can present with aggressive disease as in the case presented herein.

Undifferentiated (anaplastic) carcinomas progress rapidly and require immediate management with multimodality treatment. Neither surgery nor radiotherapy or chemotherapy is efficient alone (13).

At baseline, our patient had limited disease confined to the thyroid with no nodal involvement. The majority of patients under 45 years of age who have differentiated thyroid cancer (DTC) confined to the thyroid have an excellent prognosis (14). Clinico-pathological features that confer a poor prognosis include age over 70 years, distant metastases, lymph-node metastases >3 cm, follicular histology, and a poorly differentiated component in the primary thyroid neoplasm (15).

Interestingly, in our patient, the histopathologic findings of the lung and L4 vertebra metastases corresponded to the classical papillary component of the primary tumor in the thyroid gland and not the undifferentiated focus. This fact favors the hypothesis that, in our patient, PTC was inherently aggressive and the undifferentiated focus in the primary thyroid tumor represented the most extreme component of the same disease spectrum.

Our patient received two ablative dosages of ^131^I therapy and became ^131^I resistant with progressive elevation in Tg levels on subsequent follow-up. Thyroid carcinomas with little or no iodine activity tend to have higher glucose metabolism and positive FDG-PET scans representative of tumor dedifferentiation. Patients in this group have a higher mortality rate over 3-year follow-up as compared to patients with no FDG uptake (16). Surgery is the mainstay of management. However, in our case, radiotherapy was offered to improve local control since radioactive iodine therapy was inefficient and surgical resection of the metastases was not possible.

The primary goals for treatment at each step are to decrease morbidity from metastatic disease and to improve overall survival. Thus, local radiotherapy may be helpful in disease control in cases with potentially aggressive DTC variants, and such patients should be followed up closely.

## Ethics

Informed Consent: Consent form was filled out by all participants.

Peer-review: Externally peer-reviewed.

Financial Disclosure: The authors declared that this study has received no financial support.
